# Autoimmune sequelae in severe drug eruptions

**DOI:** 10.1186/2045-7022-4-S3-P147

**Published:** 2014-07-18

**Authors:** Yumi Aoyama, Aya Takehara, Yoshinori Shirafuji, Michiko Kurosawa, Yukiko Ushigome, Yoko Kano, Tetsuo Shiohara, Keiji Iwatsuki

**Affiliations:** 1 Okayama University, Japan; 2 Okayama University, Dermatology, Japan; 3 Juntendo University, Epidemiology and Environmental Health, Japan; 4 Kyorin University school of medicine, Dermatology, Japan

## 

It remains unknown how autoimmunity is elicited, although many different mechanisms have been involved. There is a major difficulty in establishing a correlation between triggering events and the actual autoimmune disease because of long prodromal period. In this regard, previous studies reported that sera obtained from the acute stage of SJS/TEN and erythema multiforme contain autoantibodies (autoAbs) against epidermal proteins. Unfortunately, however, it remains to be determined whether generation of these autoAbs could be a mere epiphenomenon of epidermal damage or if there could be a link between SJS/TEN and the subsequent risk of developing autoAbs and autoimmune blistering disease. Thus, there is a great need for longitudinal analyses are generated by using samples obtained at various time points including those during the acute stage of the disease and long after their clinical resolution.We retrospectively analyzed sera serially collected from 27 DiHS, 9 TEN, 30 SJS and 7 healthy controls for the presence of autoAbs against epidermal proteins such as plakins and recombinant periplakinN1-324. These autoAbs were detected in 23.3% (SJS), 55.6% (TEN) and 48.1% (DiHS) of patients, respectively. These autoAbs tended to disappear much earlier in SJS/TEN than in DiHS. Because the existence of these autoAbs was not restricted to patients with SJS/TEN but was extended to those with DiHS characterized by no epidermal damage. These results clearly indicate that those autoAbs are not the cause of epidermal damage. According to our previous study, the time-dependent defective regulatory T cell (Treg) responses occur during the acute stage (SJS/TEN) and after clinical resolution (DiHS), respectively. The defects of Treg function could provide explanation of why autoimmune responses can be generated during the acute stage(SJS/TEN)and after resolution (DiHS), respectively. Surprisingly, our analysis of the effect of systemic corticosteroids during the acute stage of DiHS on the generation of those autoAbs showed that achievement of early resolution by corticosteroids was associated with a lower risk of subsequently generating autoAbs. In conclusion, self-inflicted immune responses occurring during the acute stage of severe drug eruptions could be a trigger for the subsequent development of AutoAbs. Longitudinal analyses of these autoAbs offer the possibility for screening high-risk patients at a time before the development of overt autoimmune disease.

